# Testicular processing fluid as a useful matrix for the detection of porcine circovirus type 2 DNA and virus-specific antibodies

**DOI:** 10.3389/fvets.2026.1745725

**Published:** 2026-02-19

**Authors:** Hanna Turlewicz-Podbielska, Arkadiusz Dors, Małgorzata Pomorska-Mól

**Affiliations:** Department of Preclinical Sciences and Infectious Diseases, Faculty of Veterinary Medicine and Animal Sciences, Poznan University of Life Sciences, Poznan, Poland

**Keywords:** laboratory diagnostics, non-invasive matrices, PCV2, pig welfare, testicular processing fluid

## Abstract

**Introduction:**

Testicular processing fluid (PF) obtained during boar castration may serve as a diagnostic matrix for monitoring porcine circovirus type 2 (PCV2) presence.

**Methods:**

Anti- PCV2 antibodies in PF were detected using an indirect ELISA, and PCV2 DNA was detected by real-time PCR (qPCR), and the effects of sample pooling were evaluated. Paired sera and PF from boars and sera from gilts were tested with commercial ELISA and qPCR kits. PF-specific cut-offs were set by ROC (ELISA OD; qPCR Ct). Pooling was simulated by diluting positive PF samples with negative PF samples at predefined ratios.

**Results:**

With manufacturers’ cut-offs, seropositivity was 89.94% (male sera), 87.43% (PF), and 92.81% (gilt sera); differences were observed only between gilt sera and PF. Using the ROC PF cut-off (OD ≥ 0.23), 88.82% PF were positive, and matrices did not differ; diagnostic agreement metrics for PF improved. In qPCR, positivity was 13.02% (boar sera), 16.90% (PF), and 9.36% (gilt sera). A ROC-specific PCR cut-off (Ct < 36.50) improved specificity, predictive values, and agreement without affecting sensitivity; serum and PF Ct values were moderately correlated (*ρ* = 0.53). Pooling reduced detection of weak positives (most notably in qPCR) while high-positive PF remained detectable at higher dilutions.

**Discussion and conclusion:**

These results demonstrate that PF provides a reliable, cost effective alternative to serum for PCV2 surveillance and monitoring when matrix-specific cut-offs are used; however, excessive pooling may lead to false-negative results. This approach may facilitate large-scale herd monitoring while reducing the need for invasive sampling.

## 1 Introduction

Advancements in sampling and diagnostic techniques have simplified the collection of material for farm laboratory diagnostics. Using alternative diagnostic matrices or materials previously discarded, such as testicles after castration, for diagnostic purposes can reduce the need for additional invasive sampling (e.g., venipuncture) and increase the frequency of herd testing for routine pathogen monitoring. Early diagnosis is crucial for effectively controlling infections on farms. Utilizing samples obtained during routine farm procedures can save time, labor, and costs, ultimately improving production profitability. Reducing the need for blood collection is relevant from both an animal-handling and a logistical perspective, particularly when monitoring economically important pathogens under field conditions (1). Processing fluid (PF) constitutes a serosanguinous exudate obtained during castration and tail-docking of piglets, allowing both antibody detection and molecular detection of viral genetic material, as previously demonstrated for PRRSV (2). It represents a diagnostic sample that can be collected without additional sampling procedures (1, 2). Importantly, the diagnostic application of PF should not be interpreted as an endorsement of surgical castration; rather, its use is confined to production systems where such practices are already routinely implemented. This sample type is promising for monitoring various pathogens circulating in breeding herds and suckling piglets (2–7). Previous studies have reported the detection of the genetic material of porcine circovirus type 2 (PCV2) (7, 8) or other viruses, e.g., porcine reproductive and respiratory syndrome virus (PRRSV) (6), porcine deltacoronavirus (7), Seneca Valley virus (3) or Atypical Porcine Pestivirus (4) as well as for the detection of antibodies against PRRSV (6) or Hepatitis E virus (5). Piglet processing involves several procedures, including castration and tail docking, which are performed during the first week of life. European Union legislation prohibits routine tail docking (Council of the European Union, 2008, 2016), and measures to minimize the risk of tail biting shall be taken before practicing tail docking, e.g., provision of manipulable material (9). The exact number of pigs that undergo tail docking in Europe is currently unknown. According to Walgreen et al. (9), over 90% of pigs in the EU are tail-docked despite a ban on routine docking. However, it is worth noting that this data originates from several years ago. In the recent study by Gomes-Neves et al. (10) in Portugal, only 22% of 15,683 weaners assessed had docked tails. A decreasing trend in tail-docking is also observed in Poland. Therefore, we use the PF obtained only from boar testes in the present study. The European Declaration on alternatives to surgical castration set a goal to prohibit surgical castration by 2018 (11). However, it seems unlikely that a complete ban on surgical castration, whether with or without anesthesia, will be implemented shortly, as most European countries have not yet achieved the target outlined in the declaration. Surgical castration of male piglets remains prevalent on most commercial swine farms (12). Furthermore, concerns have been raised regarding alternative methods to castration, such as slaughtering pigs before they reach sexual maturity (13), using sperm sexing for the selection of female offspring, genetic selection for pigs with low levels of boar taint, and immunocastration (14). These concerns stem from the additional costs for farmers and uncertainties about consumer attitudes toward meat from pharmacologically castrated pigs. Additionally, the issue of a castration ban has not yet been raised in other major swine-producing countries such as China, the USA, and Brazil.

PCV2 is prevalent in nearly all commercial swine herds globally (15) and is the etiologic agent of porcine circovirus-associated diseases (PCVAD). Current understanding of porcine circovirus diseases (PCVD) caused by PCV-2 encompasses several forms, including subclinical infection (PCV-2-SI), systemic disease (PCV-2-SD), reproductive disease (PCV-2-RD), and porcine dermatitis and nephropathy syndrome (PDNS) (16). PCV2 infection commonly results in subclinical infections, which impact production parameters and cause economic losses for farmers, primarily due to reduced average daily gain (17). Despite the availability of commercial vaccines for many years, mass vaccination has not led to the eradication of the virus (18–20). Understanding the prevalence and dynamics of PCV2 infection is crucial for effectively preventing and controlling its transmission in pig herds. It is known already that PF offers a practical and cost-effective sample for PCV2 monitoring (7). However, little is known about the impact of sample dilution by PF from PCV2-negative males, which may increase the risk of obtaining false-negative results in litters with only one PCV2 antibody-positive or low viral load in only a few males. This could decrease antibody levels and viral load in pooled samples (21). For PRRSV, the effect of pooling PF samples on molecular detection has been previously evaluated and shown to strongly depend on the initial viral load and dilution level, which may substantially affect diagnostic sensitivity (22). Additionally, no previous studies have evaluated the usefulness of PF obtained only from testicles for PCV2 monitoring. Thus, the present study aimed to assess the usefulness of PF obtained only from testicles collected during male castration for PCV2 serological and molecular diagnostics, as well as the effect of sample pooling on the test results. The study also aimed to investigate the effect of sample pooling on the test results and whether the results obtained from testicular PF differ from those obtained from boar and gilt serum.

## 2 Materials and methods

### 2.1 Farms

The samples used in the study originated from 18 Polish commercial pig farms where surgical castration without tail docking was routinely performed (Council Directive 2008/120/EC). Samples from six farms in the Lubelskie Voivodeship, four farms in the Łódzkie Voivodeship, four farms in the West Pomeranian Voivodeship, and one farm each in the Opolskie Voivodeship, Masovian Voivodeship, Pomeranian Voivodeship, and Greater Poland Voivodeship were collected between August 2018 and December 2023. Only one farm in the Lubelskie Voivodeship did not implement vaccination against PCV2, and the active infection in piglets was confirmed on this farm by real-time PCR.

### 2.2 Sample collection

All samples used in this study were collected by veterinarians caring for a specific herd during routine castrations for veterinary diagnostic purposes. Samples originated from 703 piglets aged 3–5 days from 62 litters. From each litter, 1 to 15 piglets were sampled, depending on availability and herd size. Serum and testis samples were collected from each male piglet on the same day, during routine surgical castration. Blood was drawn before the procedure, and testes were collected immediately afterwards. Blood samples were collected from female piglets belonging to the same litters as the castrated males during the same farm visit. Blood samples were drawn from the vena cava cranialis into clot activator tubes (Medlab-products, Raszyn, Poland). Testes from individual male piglets were collected into 50 mL Falcon Tubes (Fisher Scientific, Hampton, USA). Prior to sample collection, field veterinarians were provided with general instructions to minimize cross- and environmental contamination during routine piglet processing, including the use of separate scalpels and gloves for each piglet. All samples were transported to a laboratory with cooling inserts. The blood was centrifuged at 2500 × g for 15 min at 4 °C to obtain serum. To obtain PF, the testes were defrosted twice at room temperature (20–22 °C) for approximately 30–60 min, depending on sample volume, until complete thawing. The serum and PF were kept frozen at −80 °C until further analysis. Individual oral informed consent was obtained from the owners for their animals’ participation in this study. Due to the limited volume of PF obtained from individual piglets, not all samples could be analyzed using both ELISA and real-time PCR. Samples with very low PF volumes were prioritized for ELISA testing, whereas samples with higher volumes were preferentially used for nucleic acid extraction and PCR analysis. Consequently, not all samples were analyzed in paired assays, which resulted in differences in sample numbers between ELISA and PCR analyses.

### 2.3 Detection of Ab against PCV2 and pooling simulation

Three hundred fifty eight male serum samples and corresponding PF samples, as well as 334 female serum samples, underwent testing using the commercial indirect ELISA kit – Ingezim Circo IgG 11. PCV. K1® (Ingenasa, Madrid, Spain). The kit is based on an indirect ELISA format using a recombinant PCV2 antigen adsorbed to the wells of microtiter plates. Samples (serum or PF) were diluted 1:200 in the dilution buffer provided with the kit (5 μL of serum and 1 mL of diluent). Then, 100 μL of each diluted sample was dispensed into individual wells and incubated at room temperature (RT, approximately 24 ± 2 °C) for 1 h. After washing three times with the supplied wash buffer, 100 μL of peroxidase-conjugated anti-pig IgG antibody was added to each well and incubated for 30 min at RT. The plates were then washed again, and 100 μL of the TMB substrate solution was added to each well. After a 10-min incubation in the dark at RT, the enzymatic reaction was stopped with 100 μL of the stop solution. The OD was measured at 450 nm with a reference wavelength of 620 nm using the Infinite 200 PRO microplate reader (TECAN, Mannedorf, Switzerland) immediately after the reaction was stopped. The manufacturer’s cut-off was calculated following the kit’s instructions (positive cut-off = OD of negative control + 0.25) for each plate. The term ELISA manufacturer’s cut-off used in the text refers to the average from individual plates. The ROC-calculated cut-off determined by ROC curve analysis was set at 0.3. Although the ELISA kit was initially validated for detecting anti-PCV2 antibodies in serum or plasma from pigs, it was not validated for use with PF. To assess the impact of pooling on the test results, the samples were categorized as low, moderate, and high-positives (OD ranges: 0.30–1.00; 1.01–2.00; 2.01–3.76, respectively). Four positive samples were randomly selected for each range. Sample pools were prepared by combining specific volumes of PCV2-positive and PCV2-negative PF to obtain pools with different dilutions (1:5, 1:10, 1:20, 1:40, and 1:80). For example, to obtain a 1:5 dilution, 50 μL of positive PF was combined with 200 μL of negative PF, resulting in a total of 250 μL of pooled sample. For a 1:10 dilution, 50 μL of positive PF was combined with 450 μL of negative PF. For higher dilutions, at least 10 μL of the positive sample was combined with an appropriate volume of the negative sample. Negative samples were selected based on the results obtained in two different ELISA kits (all samples were tested in triplicate) and subsequently used to dilute PCV2-positive PF samples to create pooled samples of varying dilutions. Pooled samples were then tested as described above.

### 2.4 PCV2 genetic material detection and pooling simulation

Three hundred sixty one pairs of sera from male piglets and PFs, along with sera from 242 female piglets, were subjected to commercial real-time PCR kit analyses. The genetic material of PCV2 was extracted from 100 μL of each serum or PF sample using the Viral DNA/RNA kit (A&A Biotechnology, Gdańsk, Poland) according to the manufacturer’s instructions and stored at −80 °C until further processing. After initial centrifugation, 100 μL of supernatant was mixed with lysis buffer and isopropanol, then applied to a spin column. The column was washed three times with washing buffer and centrifuged between each step. DNA was eluted in 60 μL of ultrapure water. To detect PCV2 DNA, we employed the ViroReal Kit PCV2 (Ingenetix GmbH, Vienna, Austria), which targets a conserved region within the ORF1 gene of the PCV2 genome. Each PCR reaction was performed in a total volume of 20 μL, consisting of 11 μL of master mix and 9 μL of DNA template. PCR amplification was performed on a Bio-Rad CFX96 Real-Time PCR Detection System. Amplification was performed under the following conditions: initial incubation at 50 °C for 2 min (Program 1), denaturation at 95 °C for 2 min (Program 2), followed by 45 cycles of denaturation at 95 °C for 5 s, annealing and extension at 60 °C for 60 s, with fluorescence data collection. A no-template control and a positive control were included in each run, provided with the kit. According to the manufacturer’s instructions, a sample was considered positive when the Ct was <45. To assess the impact of pooling on test results, we categorized samples as low, moderate, and high positives based on Ct ranges (≥36, 30.01–35.99, and ≤30, respectively) and randomly selected two positive samples from each range. Subsequently, pooled samples were prepared by combining appropriate volumes of PCV2-positive PF with PCV2-negative PF to obtain dilutions 1:10, 1:20, 1:40, 1:80, 1:160, and 1:320. For instance, to obtain a 1:10 dilution, 50 μL of positive PF was mixed with 450 μL of negative PF, yielding a 500 μL total volume. Similarly, a 1:20 dilution involved 50 μL of positive PF and 950 μL of negative PF. For higher dilutions, at least 10 μL of the positive sample was combined with an appropriate volume of the negative sample. This approach ensured accurate dilution while providing enough volume for nucleic acid extraction. DNA was extracted using the Viral DNA/RNA kit (A&A Biotechnology, Poland), which yields 60 μL of eluate per sample. PCV2-negative samples were selected based on the results obtained from real-time PCR testing of individual samples (in triplicate) and subsequently used to dilute PCV2-positive PF samples to create pooled samples of varying sizes. DNA isolation and real-time PCR were performed on each diluted sample as previously described.

### 2.5 Statistical analyses

Statistical analyses were performed between individual PF samples obtained from male piglets and paired serum samples collected from the same animals, and not at the litter or batch level. Although samples originated from 62 litters, each was treated as independent, reflecting the study’s aim to assess diagnostic performance on a population scale rather than model within-litter variation. For evaluating diagnostic agreement, serum samples were treated as the reference standard. The Chi-square test of independence was utilized to determine if there is a significant difference in the frequency of positive results in the ELISA and real-time PCR between male piglets, female piglets, and PF, with Bonferroni correction for pairwise comparisons. Due to the non-normal distribution of ELISA OD values in all tested sample types (as determined by the Shapiro–Wilk test), pairwise differences between serum from male piglets, serum from female piglets, and PF were evaluated using the Mann–Whitney *U* test (Wilcoxon rank-sum test). Sensitivity (SE), specificity (SP), negative predictive value (NPV), negative predictive value (PPV), and Cohen’s kappa coefficient (K) were computed for the method using PF instead of serum samples in commercial ELISA and real-time PCR tests. Receiver operating characteristic (ROC) analysis was used to determine the optimal cut-off value for PF in both tests. The cut-off that yielded the best possible SE and SP (closer to the top left corner of the ROC curve) was chosen. Due to the non-normal distribution of Ct values observed in both serum and processing fluid (Shapiro–Wilk test, *p* < 0.001), the relationship between paired values was assessed using Spearman’s rank correlation coefficient. The analysis included samples in which PCV2 DNA was detectable in serum, PF, or both (*n* = 67). For statistical consistency and appropriate ranking, samples with undetectable PCV2 DNA were assigned a Ct value of 45.1 (following the PCR kit manufacturer’s instruction, indicating that Ct values above 45 should be considered negative). The analyses were conducted using RStudio (version 4.5.1.) with the following packages: stats for Shapiro–Wilk normality tests, Wilcoxon rank sum tests, Spearman’s rank correlation coefficient, and Chi-squared tests; caret and epiR for generating confusion matrices and estimating diagnostic accuracy measures (SE, SP, NPV, PPV, K, and confidence intervals); ggplot2 for data visualization. The ROC curve was generated using Statistica 13.3 (Tibco, USA). The significance level was set at *α* = 0.05, and a *p*-value <0.05 was considered statistically significant.

## 3 Results

### 3.1 Detection of Ab against PCV2 and pooling simulation

**Figure 1:**
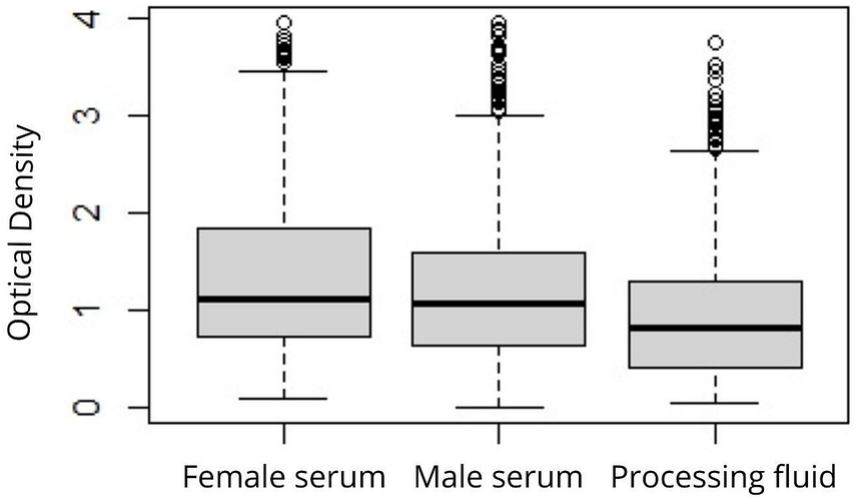
Distribution of OD values of PCV2 ELISA by sample type.

**Figure 2:**
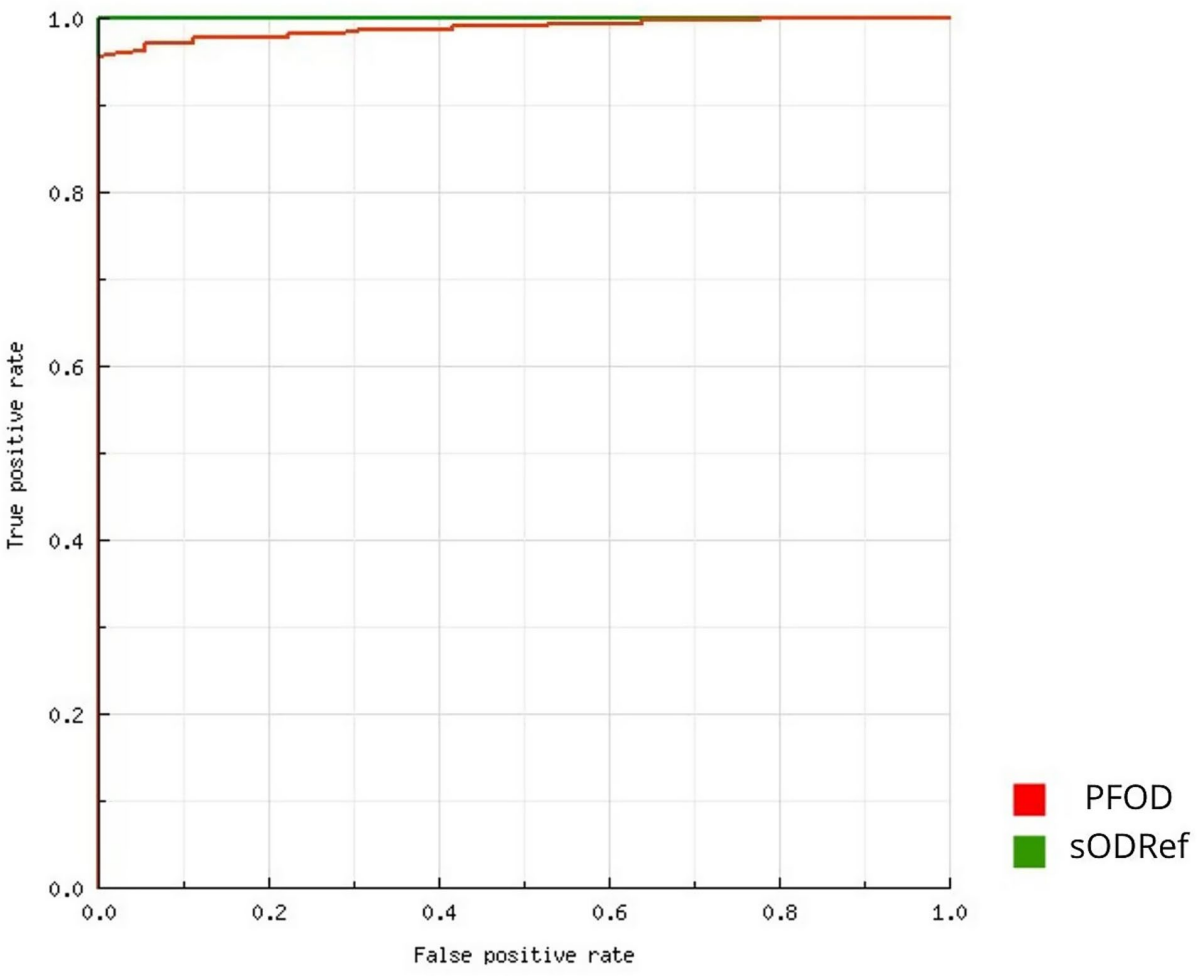
ROC curve for interpreting OD ELISA results in PF. PFOD: processing fluid OD. sODRef: male serum OD (reference).

Out of the male sera tested, 89.94% (322/358) showed positive results for anti-PCV2 antibodies. Among the corresponding PFs, 87.43% (313/358) of samples tested positive. 92.81% (310/334) of the sera tested from gilts were positive. After applying the manufacturer’s cut-off for both serum and PF samples, significant differences were observed in the percentage of positive samples between gilt sera and PF (*p* < 0.05). However, there were no significant differences in the percentage of positive samples obtained from boar sera and PF (*p* = 0.288) and between boar and gilt sera (*p* = 0.180). Table 1 presents the minimum, maximum, and average optical density values (OD). Significant differences between mean OD values of boar sera and PF (p < 0.05) and between gilt sera and PF (*p* < 0.05) were observed; however, no differences in OD values obtained from boar and gilt sera were found (*p* = 0.101) (Figure 1). The analysis of the ROC curve (Figure 2) indicated that the highest accuracy [area under the curve (AUC): 98.87%] in comparing PF and serum ELISA results was achieved when the cut-off for PF OD was ≥0.23. Using this threshold, 88.82% (318/358) of PF samples were identified as positive for PCV2-specific antibodies, and there were no significant differences in the percentage of positive samples between boar sera and PF (*p* = 0.63) and between gilt sera and PF (*p* = 0.07). Table 2 shows SE, SP, NPV, PPV, and K calculated for PF using both the manufacturer-recommended ELISA assay cut-off and the ROC-calculated cut-off values. SE, NPV, and K values for PF notably improved after the implementation of the ROC-calculated cut-off value. The contingency table presents the true positive, true negative, false positive, and false negative results of ELISA after applying the manufacturer’s cut-off and the ROC-calculated cut-off (Table 3).

**Table 1:** Minimum (min.), maximum (max.), and average OD (optical density) in the serum of females, males, and PF.

Parameter	Female sera OD	Male sera OD	PF OD
Min.	0.087	0.011	0.057
Mean	1.405^a^	1.280^a^	0.987^b^
Max	3.951	3.946	3.761

Different letters indicate statistical differences.

**Table 2:** Sensitivity (SE), specificity (SP), negative and positive predictive values (NPV, PPV), and Cohen’s Kappa coefficient (K) obtained for PF using ELISA manufacturer’s and ROC-calculated cut-off values.

Parameter	Manufacturer’s cut-off (OD^a^ ≥ 0.30)	95% CI^b^	ROC-calculated cut-off (OD ≥ 0.23)	95% CI
SE (%)	97.20 (313/322)	94.76–98.71	98.45 (317/322)	96.41–99.93
SP (%)	100.00 (36/36)	90.26–100.00	97.22 (35/36)	85.47–99.93
PPV (%)	100.00 (313/313)	98.83–100.00	99.69 (317/318)	97.87–99.95
NPV (%)	80.00 (36/45)	67.75–88.40	87.50 (35/40)	74.54–94.36
Κ	0.87	0.79–0.96	0.91	0.84–0.98

Crude numbers are shown as numerator/denominator. SE was calculated as true positives/(true positives + false negatives), SP as true negatives/(true negatives + false positives), PPV as true positives/(true positives + false positives), and NPV as true negatives/(true negatives + false negatives). All metrics were calculated for individual male PF samples using male serum as the reference method. ^a^Optical Density; ^b^Confidence interval.

**Table 3:** 2 × 2 contingency table comparing ELISA results (positive/negative) from PF and male piglet serum samples using two different cut-off values: the manufacturer-recommended cut-off and the ROC-calculated cut-off.

PF ELISA result	Manufacturer’s cut-off		ROC-based cut-off	
	Male serum positive	Male serum negative	Male serum positive	Male serum negative
PF positive	313	0	317	1
PF negative	9	36	5	35

Serum was used as the reference method.

In the next step, PF pools were tested to determine the maximum dilution and detect a single positive sample among a pool of negatives. Using the cut-off value recommended by the manufacturer, anti-PCV2 antibodies were detected in 2 out of four low-positive samples at dilutions of 1:10. Nevertheless, using the calculated cut-off, three out of four samples at dilution 1:10 and in two out of four samples at dilution 1:20 were classified as positive. In general, 25% (5/20) and 40% (8/20) of low-positive samples were correctly classified as positive after applying the manufacturer’s and ROC-calculated cut-off, respectively. Using the manufacturer’s cut-off, all moderate-positive samples were correctly classified at dilution 1:10, three out of four samples at dilution 1:20, two out of four samples at dilution 1:40 and one out of four samples at dilution 1:80. Using ROC-calculated cut-off, all moderate-positive samples were correctly classified at dilution 1:20, three out of them at dilution 1:40 and one of the at dilution 1:80. In total, 70% (14/20) and 80% (16/20) of moderate-positive samples were correctly classified as positive after applying manufacturer’s and ROC-calculated cut-off, respectively. All high-positive samples were classified correctly regardless of the cut-off used. The detailed information regarding sample classification and OD values after pooling one PF sample with an appropriate number of negative PF is presented in Table 4.

**Table 4:** Impact of pooling and dilution (1:5, 1:10, 1:20, 1:40 and 1:80) on ELISA detection of processing fluid (PF) samples with low, moderate and high optical density (OD) values.

Dilution	Low-positive samples			Moderate-positive samples			High-positive samples		
	OD^a^	Cut-off		OD	Cut-off		OD	Cut-off	
		Manufacturer’s OD ≥ 0.30	Calculated OD ≥ 0.23		Manufacturer’s OD ≥ 0.30	Calculated OD ≥ 0.23		Manufacturer’s OD ≥ 0.30	Calculated OD ≥ 0.23
Ndil^b^	0.88			1.88			2.24		
1:5	0.65	+	+	0.99	+	+	1.85	+	+
1:10	0.54	+	+	0.86	+	+	0.86	+	+
1:20	0.29	−	+	0.70	+	+	0.74	+	+
1:40	0.22	−	−	0.62	+	+	0.61	+	+
1:80	0.17	−	−	0.46	+	+	0.45	+	+
Ndil	0.80			1.74			2.21		
1:5	0.61	+	+	0.96	+	+	1.68	+	+
1:10	0.39	+	+	0.78	+	+	1.18	+	+
1:20	0.30	+	+	0.44	+	+	0.93	+	+
1:40	0.14	−	−	0.30	+	+	0.81	+	+
1:80	0.12	−	−	0.20	−	−	0.61	+	+
Ndil	0.48			1.16			2.02		
1:5	0.26	−	+	0.74	+	+	1.48	+	+
1:10	0.23	−	+	0.67	+	+	1.16	+	+
1:20	0.22	−	−	0.48	+	+	0.85	+	+
1:40	0.17	−	−	0.28	−	+	0.77	+	+
1:80	0.11	−	−	0.21	−	−	0.39	+	+
Ndil	0.39			1.13			2.01		
1:5	0.14	−	−	0.68	+	+	1.43	+	+
1:10	0.14	−	−	0.42	+	+	1.06	+	+
1:20	0.10	−	−	0.29	−	+	0.74	+	+
1:40	0.11	−	−	0.18	−	−	0.66	+	+
1:80	0.00	−	−	0.12	−	−	0.41	+	+
Correctly classified		5/20	8/20		14/20	16/20		20/20	20/20

Cut-off classification is shown as “+” (positive) and “−” (negative) according to the manufacturer’s and ROC-calculated cut-off values. ^a^Optical density; ^b^Not diluted; Low-, moderate- and high-positive samples were defined based on initial ELISA OD values as follows: low-positive, OD 0.30–1.00; moderate-positive, OD 1.01–2.00; high-positive, OD 2.01–3.76.

### 3.2 Detection of PCV2 genetic material and pooling simulation

**Figure 3:**
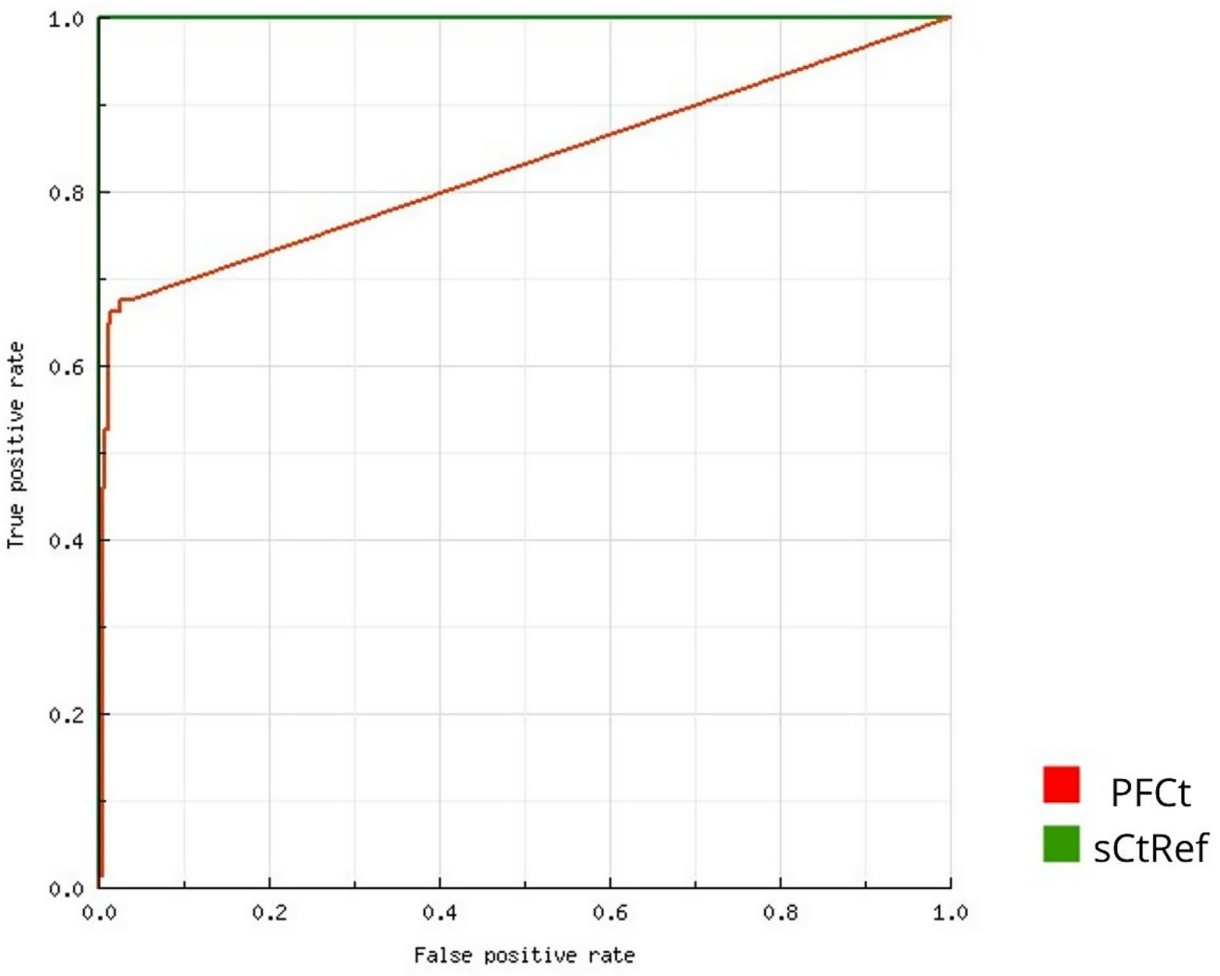
ROC curve for interpreting Ct results in PF. PFCt: Processing fluid Ct. sCtRef: Male serum Ct (reference).

**Figure 4:**
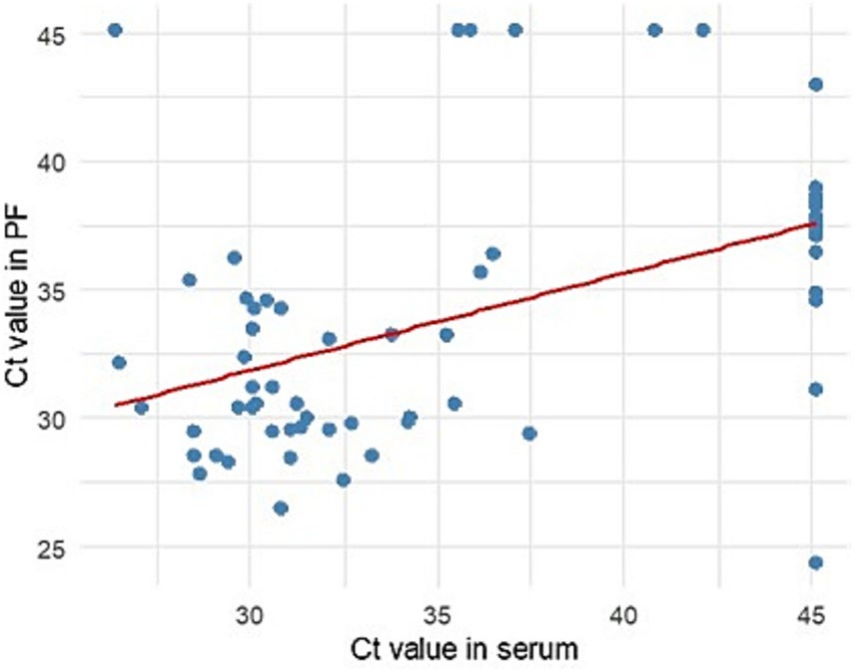
Correlation of Ct values between serum and PF. Only paired samples with detectable PCV2 DNA in at least one matrix were included in the correlation analysis.

Of the 361 male sera tested, 13.02% (47/361) were PCV2-positive. Among the corresponding PFs, 16.90% (61/361) of the samples tested positive. 9.36% (32/342) of the sera tested from gilts were positive. No significant differences in the percentage of positive samples between boar sera and PF (*p* = 0.144) and boar sera and gilt sera (*p* = 0.124) were found when using the manufacturer’s cut-off. However, a significant difference was found between gilt sera and PF (*p* < 0.05). The minimum, maximum, and average values of Ct are presented in Table 5. The ROC curve analysis (Figure 3) revealed that the best accuracy (AUC: 98.31%) between PF and serum real-time PCR results was achieved when the Ct threshold for PF was set at <36.50. Using the ROC-calculated cut-off, 12.47% (45/361) of the PF samples were classified as positive for PCV2 DNA, and no significant differences in the percentage of positive samples between boar sera, PF, and gilt sera were found (*p* = 0.267). The SE, SP, PPV, NPV, and K values calculated for PF using the cut-off recommended by the assay manufacturer and the ROC-calculated cut-off are shown in Table 6. The SP, PPV, NPV and K values for PF improved after using the new, lower cut-off point. However, the SE stayed at the same level. The contingency table presents true positive and true negative, as well as false positive and false negative results of real-time PCR after applying the manufacturer’s cut-off and the ROC-calculated cut-off (Table 7). Among 67 samples with detectable Ct values in at least one matrix (serum or PF; Supplementary material), we found a moderate and statistically significant positive correlation between serum and PF Ct values (*ρ* = 0.53, *p* < 0.05). The correlation between Ct values and serum and PF is presented in Figure 4. Samples in which PCV2 DNA was undetectable in one of the matrices were included by assigning a Ct value of 45.1, as described in the Methods.

**Table 5:** Minimum (min.), maximum (max.), and average Ct (Cycle threshold) values in the positive samples of female sera, male sera, and PF.

Parameter	Females	Males	
	Serum Ct	Serum Ct	PF Ct
Min.	26.02	26.38	24.38
Mean	31.53	31.84	32.96
Max	38.56	42.07	42.98

**Table 6:** Sensitivity (SE), specificity (SP), negative and positive predictive values (NPV, PPV), and Cohen’s Kappa coefficient (K) obtained in real-time PCR for PF using the manufacturer’s and ROC-calculated cut-off values.

Parameter	Manufacturer’s cut-off (Ct^a^ < 45)	95% CI^b^	ROC-calculated cut -off (Ct < 36.50)	95% CI
SE (%)	87.23 (41/47)	74.26–95.17	87.23 (42/47)	74.26–95.17
SP (%)	93.63 (294/314)	90.33–96.07	99.04 (311/314)	97.23–99.80
PPV (%)	67.21 (41/61)	56.95–76.06	93.33 (42/45)	81.88–97.75
NPV (%)	98.00 (294/300)	95.86–99.04	98.42 (311/316)	96.45–99.30
K	0.72	0.62–0.82	0.90	0.83–0.97

Crude numbers are shown as numerator/denominator. SE was calculated as true positives/(true positives + false negatives), SP as true negatives/(true negatives + false positives), PPV as true positives/(true positives + false positives), and NPV as true negatives/(true negatives + false negatives). All metrics were calculated for individual male PF samples using male serum as the reference method. ^a^Cycle threshold; ^b^Confidence interval.

**Table 7:** 2 × 2 contingency table comparing real-time PCR results (positive/negative) from PF and male piglet serum samples using two different cut-off values: the manufacturer-recommended cut-off and the ROC-calculated cut-off.

PF real-time PCR result	Manufacturer’s cut-off		ROC-based cut-off	
	Male serum positive	Male serum negative	Male serum positive	Male serum negative
PF positive	41	20	42	3
PF negative	6	294	5	311

Serum was used as the reference method.

Then, PF pools were tested to determine the maximum dilution that allows for the detection of one positive sample in a pool of negatives. Viral DNA was not detected in any low-positive samples, regardless of the applied cut-off after dilution. In moderate-positive samples, viral DNA was detected in one sample at a dilution of 1:80. Overall, 33.33% (4/12) and 0% (0/12) of diluted moderate-positive samples were correctly classified as positive using the manufacturer’s and ROC-calculated cut-off, respectively. PCV2 DNA was detected in all dilutions (100%; 12/12) when the manufacturer’s cut-off was used in high-positive samples. After applying the ROC-calculated cut-off, the virus’s genetic material was detectable in two samples at a dilution of 1:80 and one at a dilution of 1:320. 83.33% (10/12) of the dilutions in high-positive samples were classified correctly after using the ROC-calculated threshold. Detailed information regarding sample classification and Ct values after pooling one PF sample with an appropriate number of negative PF samples is presented in Table 8.

**Table 8:** Impact of pooling and dilution (1:5, 1:10, 1:20, 1:40, 1:80, 1:160, and 1:320) on real-time PCR detection of processing fluid (PF) samples with low, moderate and high viral load.

Dilution	Low-positive samples			Moderate-positive samples			High-positive samples		
	Ct^a^	Cut-off		Ct	Cut-off		Ct	Cut-off	
		Manufacturer’s Ct < 45	Calculated Ct < 36.50		Manufacturer’s Ct < 45	Calculated Ct < 36.50		Manufacturer’s Ct < 45	Calculated Ct < 36.50
Ndil^b^	38.29			35.46			23.04		
1:10	−	−	−	−	−	−	26.98	+	+
1:20	−	−	−	−	−	−	27.69	+	+
1:40	−	−	−	−	−	−	28.62	+	+
1:80	−	−	−	−	−	−	31.13	+	+
1:160	−	−	−	−	−	−	31.00	+	+
1:320	−	−	−	−	−	−	33.97	+	+
Ndil	37.85			35.17			30.00		
1:10	−	−	−	37.51	+	−	32.82	+	+
1:20	−	−	−	37.80	+	−	33.15	+	+
1:40	−	−	−	38.14	+	−	35.95	+	+
1:80	−	−	−	38.77	+	−	36.10	+	+
1:160	−	−	−	0	−	−	37.11	+	−
1:320	−	−	−	0	−	−	38.19	+	−
Correctly classified		0/12	0/12		4/12	0/12		12/12	10/12

Cut-off classification is shown as “+” (positive) and “−” (negative) according to the manufacturer’s and ROC-calculated cut-off values. ^a^Ct – Cycle threshold; ^b^Not diluted; Low-, moderate- and high-positive samples were defined based on initial Ct values as follows: low-positive, ≥36; moderate-positive, 30.01–35.99; high-positive, ≤30.

## 4 Discussion

This study assessed the suitability of testicular PF as a valuable sample for detecting anti-PCV2 antibodies and PCV2 genetic material. The study utilized a commercially available ELISA, initially designed for serum samples, and a commercial real-time PCR assay, originally developed for serum, urine, feces, and tracheobronchial swabs, to identify anti-PCV2 antibodies and PCV2 DNA, respectively, in PF samples. Serum samples from female piglets were included in the analyses to determine whether there were differences between boar and gilt serum results, as well as PF and serum female results. Most previously published studies on PF have analyzed samples collected during piglet processing that included both castration and tail docking. Consequently, such PF represents a composite sample derived from testes and tails. In contrast, the present study focused exclusively on processing fluid obtained from testes during male castration. This distinction may influence sample composition and representativeness and should be considered when comparing results across studies and extrapolating findings from testicular processing fluid to conventional processing fluid collected at the litter or batch level.

In the case of PCV2, most commercial swine herds are seropositive, resulting in efficient transfer of PCV2-specific antibodies from vaccinated or naturally exposed sows to their offspring via colostrum (23). Moreover, once the fetus becomes immunocompetent, from approximately day 70 of gestation, in utero exposure to PCV2 may result in the birth of healthy seropositive piglets (24, 25). Most samples, both from porcine serum and PF, originated from herds that had undergone vaccinations. Hence, these samples tested positive for anti-PCV2 antibodies, likely due to the passive transfer of maternal antibodies via colostrum. The serological analyses revealed a significant difference in the OD values between PF and the serum. We found a notable contrast in the percentage of positive samples between gilt sera and PF when using the manufacturer’s cut-off. It is important to consider that applying the results obtained from the testicular PF to the entire group of tested animals, including gilts, could lead to an inaccurate epidemiological assessment of the herd regarding PCV2. Upon reducing the cut-off from 0.30 to 0.23, no statistically significant difference in the percentage of positive samples was found between gilts and PF. It implies the potential acceptability testing of PF derived only from boar testicles to evaluate the health status of the entire sampled animal cohort, including gilts. This adjustment improved the method’s validation parameters, such as SP, NPV, and K value, but slightly decreased SE. This means that while applying an ROC-optimized lower cut-off may improve sensitivity and reduce the number of false negatives, it may also slightly increase the risk of false positives, depending on the characteristics of the ELISA. Our findings align with another study on PF, which utilized a commercial ELISA to detect antibodies against the Hepatitis E virus in piglets. This study also found that recalculating the cut-off to a lower value improved the agreement between serum and PF (5).

Pooling experiments were performed as a proof-of-concept to illustrate the effect of dilution on PF-based ELISA and real-time PCR detection and were not intended as a quantitative population-level assessment. Upon examination of pooled samples, it was found that the final result was influenced by the initial concentration of antibodies in a single positive sample. The utilization of the ROC-calculated cut-off value reduced the number of false-negative pooled samples within low- and moderate-positive samples. In the low-positive category, 25 and 40% of samples were classified as positive using the manufacturer’s and ROC-calculated cut-off, respectively. In the moderate-positive category, 60 and 80% of samples were accurately classified using the manufacturer’s and ROC-calculated cut-off, respectively. Altering the cut-off did not influence the outcomes within the high-positive group. The ELISA must exhibit sufficient sensitivity to identify at least one positive animal in a dilution of around 1:20 when testing samples from farrowing pens, usually containing up to 20 piglets. Following the recalibration of the cut-off value, one positive sample was successfully detected amidst 19 negative samples within all moderate and high-positive pools in our study. Overall, 44 diluted samples were accurately classified using the new threshold, compared to 37 samples classified using the manufacturer’s threshold, out of the 60 diluted samples. These outcomes are consistent with a similar study by Di Bartolo et al. (5) about detecting anti-Hepatitis E antibodies in PF pooled samples. Based on the results of the present study, it is worth noting that pooling PF samples may lead to an underestimation of herd-level seroprevalence of PCV2, particularly in cases with low or moderate antibody levels, due to dilution effects.

Several studies have highlighted the reliability of PFs for molecular detection of PCV2 or other porcine viruses (3, 4, 6, 8). The present study noted a significant disparity in the percentage of PCR-positive samples between female serum and PF when employing the manufacturer’s cut-off. However, as these samples originate from different animal populations, this comparison should be interpreted with caution. Importantly, no significant differences were observed between PCR results obtained from male serum and corresponding PF samples. In addition, the proportion of PCR-positive animals did not differ significantly between male and female piglets across the PCV2-positive farms included in this study. The application of the ROC-optimized threshold reduced the discrepancy observed between female serum samples and PF results, indicating that this difference was primarily related to matrix-specific diagnostic performance rather than true population-level variation. The analysis also revealed that the real-time PCR test conducted on PF exhibited a high sensitivity (SE) of 87.23% and specificity (SP) of 93.63%, with a substantial agreement between PF and male sera (*K* = 0.72). Upon implementing the new threshold (Ct < 36.50), we observed an enhancement in SE (93.63 to 98.04%) and an improvement in *K* value (0.72 to 0.90) between these two sample types. The threshold point determined by the ROC curve bolstered the validation parameters of the method utilizing PF as a diagnostic sample (SE, PPV, NPV, and K). Notably, the SE of the method remained unchanged, irrespective of the chosen threshold. It is worth mentioning that using PF as a diagnostic sample may not identify all true positive samples, particularly from piglets with high Ct values in serum. Therefore, the outcomes of real-time PCR utilizing PF for PCV2 genetic material identification should be carefully interpreted. Using the manufacturer’s cut-off, we observed 20 positive PF from pigs with negative results in serum. These differences were reduced after applying the ROC-calculated cut-off. We assessed the correlation between Ct values in serum and PF to explore the relationship between viral loads in the two matrices. This analysis revealed a moderate, statistically significant positive correlation between Ct values in serum and PF, suggesting a consistent relationship across matrices despite some variability. Notably, PF samples generally exhibited lower Ct values than paired serum samples, indicating higher viral loads and reinforcing the potential of PF as a more sensitive diagnostic matrix for PCV2 detection at the individual or herd level.

Other studies have also detected PCV2 more frequently in matrices other than serum. Woźniak et al. (26) detected PCV2 DNA more often in oral fluid in 46.1% (82/178) of the samples from animal farms and 64.5% (20/31) from non-vaccinated farms. For comparison, the detection rate of PCV2 in serum was 17.6% (54/307) in vaccinated farms and 63.5% (33/52) in non-vaccinated farms. Researchers point out that PCV2 genetic material in oral fluid may indicate environmental contamination. Therefore, the oral fluid captures environmental PCV2 contamination within the pen and the contribution of multiple animals, which may partly explain higher detection rates compared with serum and limit its direct interpretation as an indicator of individual infection status. Similar results were observed by Nielsen et al. (27). The authors collected 310 serum pools and oral fluid sample pairs from two herds, where 4–5 pigs were bled per pen, and where 5–33 pigs lived per pen. In the first herd, 80% of serum pools and 86% of oral fluid samples were positive for PCV2. In the second herd, 91% of serum pools and 100% of oral fluid samples were positive for PCV2. According to the author’s assumptions, a slightly higher proportion of viral DNA-positive oral fluid samples compared to viral DNA-positive serum could be found due to contamination of the rope with PCV2 present in feces. Moreover, the likelihood of a positive result is probably higher when more animals are tested (4 or 5 pigs were bled versus up to 33 pigs contributing to the oral fluid sample (27)). Recently, Igriczi et al. (28) observed the highest detection rates of PCV3 in the PF samples compared to those in serum. However, the mentioned study focused on different types of porcine circovirus, and the samples of PF were collected from a large group of animals – one plastic bag contained testicles and fluids of approximately 10 litters. In contrast, our study tested individual PF samples. In the study by Igriczi et al. (28), the overall proportion of PCV3-positive samples was 61% (59/97) in the positive farms. The detection frequencies were lower in the oral fluid samples, as the PCV3 was detected in 40% (71/176) of the samples. What is aligned with the above studies is that the lowest PCV3 detection rate was observed in the serum pools (23%; 85/371). The Ct values of the PF samples in the mentioned study were significantly lower than in the sera and oral fluid samples, with a mean value of 30.18 (28). Finally, Eddicks et al. (29) reported a high PCV2 detection rate in tissues (inguinal lymph node, spleen, thymus, lung, and myocardium) obtained from piglets crushed by their dams, with 61.6% of the 185 crushed piglets from 134 litters being PCV2 DNA-positive. However, the same research team, in earlier studies, did not detect PCV2 DNA in any of the serum samples from piglets before or after colostrum intake. All 590 pre-suckling and 581 post-suckling blood samples were PCV2 DNA-negative (30). The same results were obtained by Diste-Perez et al. (31). None of the 352 blood samples from the live-borne piglets analyzed resulted positively in the real-time PCR (31). Finally, our study aligns with the results obtained by Fan et al. (8), who found a higher detection rate of PCV2 genetic material in PF samples from pre-weaning piglets (25.7%; 118/459) compared to serum (1.2%; 5/404). Fan et al. (8) suggest that high positivity of testicular fluids indicates that infection occurs early in piglets, and the risk of PCV2-associated diseases is high after weaning. Additional support for the diagnostic relevance of testicle-derived matrices comes from a recent study by Fan et al. (29). In that study, testicles from approximately 20 litters were pooled, resulting in 415 pooled samples, of which 31.1% were positive for PCV2 DNA by real-time PCR. Unlike the present work, studies by Fan et al. (8, 32) were performed at litter-level. These findings suggest that testicular PF may be a more effective means of monitoring PCV2 than other matrices (8). The higher frequency of PCV2 DNA detection in PF samples compared with serum observed in the present study may be related to differences in matrix sensitivity and to the temporal and anatomical distribution of PCV2 early after birth. PF-positive/serum-negative pairs may therefore reflect matrix-specific detection characteristics rather than true disagreement in infection status. After birth, PCV2 replication occurs mainly in lymphoid tissues, although viral DNA has also been detected in epithelial and endothelial cells (33–35). In addition, cells of the monocytic lineage may accumulate PCV2 genetic material for prolonged periods without productive infection (36, 37), which may result in detection of viral DNA in tissue-associated matrices in the absence of detectable viremia. PCV2 DNA has previously been detected in tissues of neonatal piglets despite negative serum results (29, 30), and intermittent shedding in the male reproductive tract has also been reported (38–40). Evidence supporting the presence of PCV2 in reproductive tissues has also been reported outside commercial pig production. In a recent study conducted in southern Italy, PCV2 DNA was detected in 5 out of 37 reproductive tissue samples collected from wild boars, including testes from males and uterine tissues from females, using molecular methods (41). In addition, detection of PCV2 DNA in PF samples in this study may partly reflect environmental contamination. Although measures were taken to minimize cross-contamination during sampling, this possibility cannot be fully excluded. Conversely, serum-positive/PF-negative results may occur when viremia is present at low levels near the assay detection limit. Further studies are needed to clarify the biological basis of discordant PF and serum results. Using PF may be a convenient way to verify the presence of PCV2 in a given herd and to assess PCV2 occurrence in newborn piglets, where the virus is implicated in various pathologies. Nevertheless, the results from PF in the context of the presence of PCV2 genetic material should be interpreted with caution until further research using other techniques confirms the presence of PCV2 in testis tissue. It is important to note that PF was collected after two freeze–thaw cycles in this study, which differs from typical field practice. The second thawing was necessary to obtain sufficient fluid volume for analysis at the individual sample level. While PCV2 DNA is considered stable under frozen conditions (42), repeated freeze-thawing may compromise nucleic acid quality, and this procedural difference should be taken into account when interpreting the results.

When pooling samples and subjecting them to molecular analyses, we noted that the initial Ct of the diluted sample influences the outcome. Additionally, fewer tested samples were accurately identified as positive after implementing the ROC-calculated cut-off. In a study conducted by Lopez et al. (22), it was observed that the accuracy in correctly classifying a sample as positive was 95% within a cohort comprising 28 PRRSV-negative litters and one PRRSV-positive litter, which included 323 piglets. The authors’ regression analysis model for the serially diluted processing fluid sample suggests that confining the processing fluid sampling to 30 litters or fewer (approximately 320 piglets) is anticipated to yield a 95% confidence level in detecting PRRSV RNA, even when the prevalence within piglets is minimal (22). In contrast to PRRSV, extrapolating pooled PF results to estimate herd-level epidemiological status for PCV2 warrants careful consideration, especially in scenarios characterized by low viral loads and elevated dilution ratios, where the accuracy of such inferences may be significantly compromised. In our study, none of the diluted samples in the low-positive group were correctly identified, regardless of the threshold. In the moderate-positive group, the percentage of correctly identified samples ranged from 0 to 33%, depending on the cut-off value used. Among the high-positive samples, the percentage of samples correctly identified as positive ranged from 83.33 to 100% for the ROC-calculated and standard cut-off, respectively. It is important to note that higher accuracy depends on having a low Ct value in the initial positive PF sample. The probability of PCV2 RNA detection in a pooled sample containing one sample from a PCV2-positive piglet decreases with increasing dilution. As with serological results, molecular testing of pooled PF samples will not provide reliable information regarding the PCV2 epidemiologic situation in a given herd.

This study has several limitations that should be acknowledged. PF sampling is inherently linked to routine piglet processing procedures and is therefore applicable only in production systems that perform castration. Moreover, PF provides a single early-life sampling point and does not allow longitudinal monitoring of individual animals, which limits its use as a stand-alone tool for dynamic follow-up diagnostics. Consequently, PF-based testing should be interpreted primarily as a tool for early-life and herd-level surveillance rather than repeated individual diagnostics. In addition, the biological mechanisms underlying PCV2 presence in testicular-derived PF, including tissue distribution and viral kinetics in neonatal piglets, remain incompletely understood and require further experimental investigation. Finally, serum was used as the reference matrix in this study; however, biological compartmentalization between blood and tissue-derived fluids may inherently influence diagnostic concordance. Therefore, PF should be regarded as a complementary, context-specific diagnostic matrix rather than a universal surveillance tool. Another limitation of this study is the lack of complete farm-level metadata, including detailed PCV2 vaccination protocols, sow parity, and information on repeated farm visits. Samples were collected by field veterinarians during routine piglet processing procedures. Due to strict biosecurity measures related to African swine fever in Poland and the retrospective nature of sample submission, comprehensive production and management data could not be consistently obtained for all herds. Therefore, the potential impact of these factors on PCV2 detection in PF could not be evaluated and should be addressed in future prospective studies.

Although PF is intrinsically limited to male piglets, comparison with serum samples from females allowed to evaluate whether testicular PF-based results reflect PCV2 circulation at the population level, rather than to imply biological equivalence of male and female reproductive tissues. Lack of discernible differences between boars and gilts in the tests conducted on serum samples from both genders, and PF obtained exclusively from the testes may serve as a practical biological material for PCV2 circulation within breeding herds. A lower cut-off point should be applied to enhance the validation parameters of the ELISA and PCR tests dedicated to serum, but used for PF. It is important to note that the findings regarding the suboptimal performance of PF as a diagnostic matrix are based on results from a single commercial ELISA and PCR kit. Therefore, any recommendation for cut-off revision should be treated with caution and may not be generalizable across different test kits. Caution should be taken in interpreting the results of PF concerning the presence of PCV2 DNA, and further research is required in this field. Moreover, testing of pooled PF samples may lead to an inaccurate assessment of the herd’s epidemiological situation. One potential limitation of this study is that some samples originated from the same litters, which could introduce intra-litter correlation. However, our analysis was conducted at the individual-sample level, and the aim was not to assess within-litter infection dynamics, but rather to evaluate whether PF could serve as a reliable alternative to serum for population-level PCV2 detection. Therefore, although the litter effect may exist, it was not accounted for in the statistical models, which aligns with the study’s conceptual framework.

## 5 Conclusion

This study demonstrated that PF obtained from testicles can serve as a valuable alternative diagnostic matrix for detection of PCV2 at the herd level. Adjusting the diagnostic cut-off improved the agreement between ELISA and real-time PCR results obtained from PF and serum, suggesting that PF may provide reliable information on herd exposure status when properly validated. However, pooling PF samples can significantly underestimate the true prevalence of PCV2 due to dilution effects, especially in samples with low antibody titers or viral loads. Therefore, pooled PF testing should be interpreted with caution, and individual PF testing is preferable for accurate PCV2 detection. The findings also indicate no significant differences between results obtained from boar and gilt serum, supporting the feasibility of using testicular PF as a representative sample for herd-level monitoring. Nevertheless, the current conclusions are based on a single commercial ELISA and PCR kit, and further validation with other diagnostic systems is necessary before routine implementation. Overall, PF provides an alternative diagnostic matrix obtained without additional sampling procedures. That may complement existing diagnostic tools for PCV2 surveillance and contribute to improving biosecurity and monitoring practices within pig production systems.

## Data Availability

The original contributions presented in the study are included in the article/Supplementary material, further inquiries can be directed to the corresponding author.
