# Supplementary material for: Testicular processing fluid as a useful matrix for the detection of porcine circovirus type 2 DNA and virus-specific antibodies
Source: Front Vet Sci. 2026 Feb 19;13:1745725. doi: 10.3389/fvets.2026.1745725 (PMC12960188; doi:10.3389/fvets.2026.1745725)
Supplement: Supplementary file 1 [file Table_1.DOCX]

Supplementary Material

# Supplementary Table

Comparison of Ct values obtained from serum and PF samples collected from the same piglets (n = 67).

| Sample | Serum Ct^a^ | PF Ct | Sample | Serum Ct | PF Ct | Sample | Serum Ct | PF Ct |
| --- | --- | --- | --- | --- | --- | --- | --- | --- |
| 1 | 32.45 | 27.56 | 2 | 29.81 | 32.41 | 3 | 31.24 | 30.54 |
| 4 | 37.45 | 29.41 | 5 | 36.45 | 36.41 | 6 | 32.11 | 29.55 |
| 7 | 26.45 | 32.11 | 8 | 30.11 | 34.25 | 9 | 35.43 | 30.56 |
| 10 | 30.03 | 33.45 | 11 | 28.34 | 35.41 | 12 | 30.12 | 30.56 |
| 13 | 35.21 | 33.21 | 14 | 30.77 | 34.28 | 15 | 31.34 | 29.67 |
| 16 | 27.04 | 30.44 | 17 | 35.55 | NEG | 18 | 36.13 | 35.72 |
| 19 | 33.76 | 33.24 | 20 | 32.66 | 29.76 | 21 | 29.08 | 28.56 |
| 22 | 30.06 | 30.41 | 23 | 28.45 | 29.46 | 24 | 29.05 | 28.53 |
| 25 | 31.48 | 30.05 | 26 | 33.20 | 28.56 | 27 | 29.67 | 30.45 |
| 28 | 30.04 | 31.22 | 29 | 29.87 | 34.67 | 30 | 32.11 | 33.11 |
| 31 | 35.89 | NEG^b^ | 32 | 29.54 | 36.22 | 33 | 30.41 | 34.56 |
| 34 | 31.05 | 28.45 | 35 | 34.22 | 29.88 | 36 | 30.56 | 31.22 |
| 37 | 37.04 | NEG | 38 | 31.06 | 29.55 | 39 | 30.77 | 26.45 |
| 40 | 28.45 | 28.56 | 41 | 29.41 | 28.33 | 42 | 30.56 | 29.51 |
| 43 | 34.23 | 30.05 | 44 | 28.63 | 27.85 | 45 | NEG | 37.77 |
| 46 | NEG | 37.49 | 47 | NEG | 38.38 | 48 | 42.07 | NEG |
| 49 | NEG | 37.85 | 50 | NEG | 42.98 | 51 | NEG | 38.43 |
| 52 | NEG | 34.61 | 53 | 26.38 | NEG | 54 | NEG | 37.30 |
| 55 | 40.81 | NEG | 56 | NEG | 34.93 | 57 | NEG | 36.51 |
| 58 | NEG | 37.51 | 59 | NEG | 38.97 | 60 | NEG | 37.12 |
| 61 | NEG | 37.38 | 62 | NEG | 38.97 | 63 | NEG | 38.29 |
| 64 | NEG | 37.67 | 65 | NEG | 38.69 | 66 | NEG | 24.38 |
| 67 | NEG | 31.1 |  |  |  |  |  |  |

^a^Ct – Cycle threshold; NEG – Negative sample

**
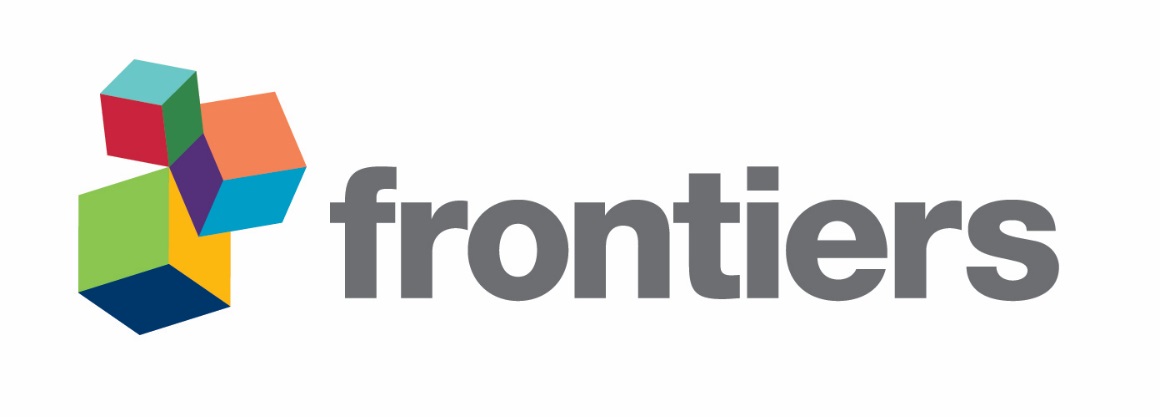
**
